# 6,6′-Dimeth­oxy-2,2′-[(*E*,*E*′)-(4-chloro-*m*-phenyl­ene)bis­(nitrilo­methyl­idyne)]diphenol

**DOI:** 10.1107/S1600536809037386

**Published:** 2009-09-30

**Authors:** Bohari M. Yamin, Siti Najihah A. Bakar, Karimah Kassim, Hadariah Bahron

**Affiliations:** a School of Chemical Sciences and Food Technology, Univeriti Kebangsaan Malaysia, UKM 43500 Bangi Selangor. Malaysia; b Department of Chemistry, Faculty of Applied Sciences, Universiti Teknologi MARA, 40450 Shah Alam, Selangor, Malaysia

## Abstract

The title compound, C_22_H_19_ClN_2_O_4_, has the appearance of a warped butterfly. One 2-hydr­oxy-3-methoxy­benzyl­idene­amino fragment is planar [with a maximum deviation of 0.056 (3) Å] and forms a dihedral angle of 9.85 (9)° with the central benzene ring. The other fragment is not planar; however, the methoxy­phenol group is planar [with the maximum deviation of 0.033 (2) Å] and makes a dihedral angle of 41.7 (3)° with the central benzene ring. The mol­ecule is stabilized by intra­molecular O—H⋯N hydrogen bonding. The crystal structure is stabilized by weak inter­molecular C—H⋯O hydrogen bonding and C—H⋯π inter­actions.

## Related literature

For the biological activity of Schiff bases, see: Aranha *et al.* (2007 ▶) and for the corrosion inhibition potential of Schiff bases, see: Chetouani *et al.* (2005 ▶). For related structures, see: Hernández-Molina *et al.* (1997 ▶); Torayama *et al.* (1997 ▶). For bond-length data, see: Allen *et al.* (1987 ▶).
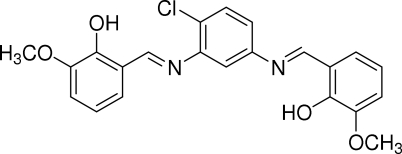


## Experimental

### 

#### Crystal data

C_22_H_19_ClN_2_O_4_
*M* *_r_* = 410.84
Monoclinic, 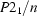

*a* = 9.900 (2) Å
*b* = 6.8589 (12) Å
*c* = 28.830 (6) Å
β = 94.659 (4)°
*V* = 1951.2 (7) Å^3^
*Z* = 4
Mo *K*α radiation
μ = 0.23 mm^−1^
*T* = 298 K
0.41 × 0.40 × 0.11 mm

#### Data collection

Bruker SMART APEX CCD area-detector diffractometer
Absorption correction: multi-scan (*SADABS*; Bruker, 2000 ▶) *T*_min_ = 0.912, *T*_max_ = 0.975
12204 measured reflections
4049 independent reflections
2758 reflections with *I* > 2σ(*I*)
*R*_int_ = 0.030

#### Refinement

*R*[*F* ^2^ > 2σ(*F* ^2^)] = 0.055
*wR*(*F* ^2^) = 0.131
*S* = 1.06
4049 reflections
272 parameters
2 restraints
H atoms treated by a mixture of independent and constrained refinement
Δρ_max_ = 0.21 e Å^−3^
Δρ_min_ = −0.17 e Å^−3^

### 

Data collection: *SMART* (Bruker, 2000 ▶); cell refinement: *SAINT* (Bruker, 2000 ▶); data reduction: *SAINT*; program(s) used to solve structure: *SHELXS97* (Sheldrick, 2008 ▶); program(s) used to refine structure: *SHELXL97* (Sheldrick, 2008 ▶); molecular graphics: *SHELXTL* (Sheldrick, 2008 ▶); software used to prepare material for publication: *SHELXTL*, *PARST* (Nardelli, 1995 ▶) and *PLATON* (Spek, 2009 ▶).

## Supplementary Material

Crystal structure: contains datablocks global, I. DOI: 10.1107/S1600536809037386/dn2481sup1.cif

Structure factors: contains datablocks I. DOI: 10.1107/S1600536809037386/dn2481Isup2.hkl

Additional supplementary materials:  crystallographic information; 3D view; checkCIF report

## Figures and Tables

**Table 1:** Hydrogen-bond geometry (Å, °)

*D*—H⋯*A*	*D*—H	H⋯*A*	*D*⋯*A*	*D*—H⋯*A*
O2—H2⋯N1	0.83 (3)	1.81 (3)	2.586 (3)	155 (3)
O4—H4⋯N2	0.82 (3)	1.86 (3)	2.588 (3)	148 (3)
C11—H11⋯O4^i^	0.93	2.59	3.392 (3)	145
C3—H3⋯*Cg*3^ii^	0.93	2.89	3.635 (3)	138

Symmetry codes: (i) 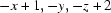; (ii) 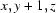. *Cg*3 is the centroid of the C16–C21 ring.

**Fig. 1.**
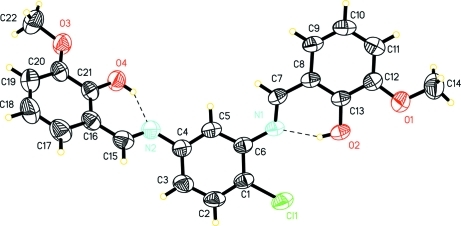
Molecular structure of compound, (1), with displacement ellipsoid drawn at the 50% probablity level. H atoms are represented as small sphere of arbitrary radii.

## supplementary crystallographic information

### Comment

Continous studies on Schiff bases are driven by their biological activities such as antimicrobial (Aranha *et al.*, 2007) and chemical properties such as corrosion inhibition (Chetouani *et al.*, 2005). Some *m*-phenylenediamine derived Schiff bases such as *N*,*N*'-disalicylidene-1,3-diiminobenzene and their complexes have been reported (Hernández-Molina *et al.*, 1997; Torayama *et al.*, 1997). The present compound is also a *m*-phenylenediamine derived Schiff base but with a chloro substituent at the *ortho* position. The Schiff base groups that attached to the 1,3-positions are 2-iminomethyl-6-methoxyphenols (Fig.1).

The whole molecule appears like a warped butterfly. The 2-imino methyl-6-methoxyphenol right wing N1/C7—C14)/O1/O2 is planar with a maximum deviation of 0.056 (3)Å for C14 atom from the least square plane. However, the left wing is twisted with the C15—N2—C4—C3 torsion angle of 41.7 (3)° compared to 9.1 (3)°. for the C7—N1—C6—C5 torsion angle of the right wing. The methoxyphenol O3/O4/(C16—C22) fragment of the left wing is planar with the maximum deviation of 0.033 (2)Å for C20 atom. As a result, the methoxyphenol groups are in opposite orientation. The central (C1—C6) benzene ring makes dihedral angle of 9.85 (9)Å with the right N1/C7—C14)/O1/O1 wing and 44.25 (9)° with the O3/O4/(C16—C22) methoxyphenol fragment. The dihedral angle between the right wing and the methoxyphenol fragment is 53.17 (7)°. The bond lengths and angles are in normal ranges (Allen *et al.*, 1987).

There are two O—H···N intramolecular hydrogen bonds (Table 1). In the crystal structure, the molecule is stabilized by weak C—H···O intermolecular hydrogen bonds, C—H···π interaction (Tables 1) and van der Waal forces.

### Experimental

The compound was synthesized by refluxing 1,3-diamino-4-chlorobenzene (0.428 g, 3 mmol) with 3-methoxysalicylaldehyde (0.912 g, 6 mmol) in ethanol for 24 h. The precipitate obtained was filtered off, washed with ethanol and dried in-vacuo. It was recrystallized from a mixed solvent of chloroform and ethanol (1:1) to afford brownish yellow single crystals. Yield 92%. Melting point 468–470 K. Analytical calculation for C_22_H_19_ClN_2_O_4_ [Cl-mpd(*o*-van)_2_]: C, 64.31; H, 4.66; N, 6.82. Found: C, 64.18; H, 4.65; N, 6.93. IR (cm^-1^): ν(C=N) 1611.7 (*m*), ν(C—O—C) 1253.9 (*s*), ν(C—OH) 1212.7 (*w*), ν(C—Cl) 1099.8 (*w*). ^1^H NMR (CDCl_3_, 300 MHz, p.p.m.): δ = 13.5002 (1*H*, s, OH), 13.2536 (1*H*, s, OH), 8.737 (1*H*, s, HC=N), 8.684 (1*H*, s, HC=N), 7.220–6.935)(9*H*, m, H-aromatic), 3.977 (3*H*, s, OCH_3_), 3.969 (3*H*, s, OCH_3_).

### Refinement

H atoms on C were positioned geometrically with C—H 0.93, 0.96 Å, for aromatic and methyl H atoms respectively, and constrained to ride on their parent atoms with *U*_iso_(H)= *xU*_eq_(C) where *x*=1.5 for methyl H and *x*=1.2 for aromatic H atoms. The H atom attached to oxygen atoms were located from the Fourier difference map and refined isotropically.

### Crystal data

**Table:**

C_22_H_19_ClN_2_O_4_	*F*(000) = 856
*M**_r_* = 410.84	*D*_x_ = 1.399 Mg m^−^^3^
Monoclinic, *P*2_1_/*n*	Melting point = 468.0–470.0 K
Hall symbol: -P 2yn	Mo *K*α radiation, λ = 0.71073 Å
*a* = 9.900 (2) Å	Cell parameters from 2429 reflections
*b* = 6.8589 (12) Å	θ = 1.4–26.5°
*c* = 28.830 (6) Å	µ = 0.23 mm^−^^1^
β = 94.659 (4)°	*T* = 298 K
*V* = 1951.2 (7) Å^3^	Block, yellow
*Z* = 4	0.41 × 0.40 × 0.11 mm

### Data collection

**Table:**

Bruker SMART APEX CCD area-detector diffractometer	4049 independent reflections
Radiation source: fine-focus sealed tube	2758 reflections with *I* > 2σ(*I*)
graphite	*R*_int_ = 0.030
Detector resolution: 83.66 pixels mm^-1^	θ_max_ = 26.5°, θ_min_ = 1.4°
ω scans	*h* = −12→12
Absorption correction: multi-scan (*SADABS*; Bruker, 2000)	*k* = −8→7
*T*_min_ = 0.912, *T*_max_ = 0.975	*l* = −29→36
12204 measured reflections	

### Refinement

**Table:**

Refinement on *F*^2^	Primary atom site location: structure-invariant direct methods
Least-squares matrix: full	Secondary atom site location: difference Fourier map
*R*[*F*^2^ > 2σ(*F*^2^)] = 0.055	Hydrogen site location: inferred from neighbouring sites
*wR*(*F*^2^) = 0.131	H atoms treated by a mixture of independent and constrained refinement
*S* = 1.06	*w* = 1/[σ^2^(*F*_o_^2^) + (0.0536*P*)^2^ + 0.4146*P*] where *P* = (*F*_o_^2^ + 2*F*_c_^2^)/3
4049 reflections	(Δ/σ)_max_ = 0.002
272 parameters	Δρ_max_ = 0.21 e Å^−^^3^
2 restraints	Δρ_min_ = −0.17 e Å^−^^3^

### Special details

**Table:**

Geometry. All e.s.d.'s (except the e.s.d. in the dihedral angle between two l.s. planes) are estimated using the full covariance matrix. The cell e.s.d.'s are taken into account individually in the estimation of e.s.d.'s in distances, angles and torsion angles; correlations between e.s.d.'s in cell parameters are only used when they are defined by crystal symmetry. An approximate (isotropic) treatment of cell e.s.d.'s is used for estimating e.s.d.'s involving l.s. planes.
Refinement. Refinement of *F*^2^ against ALL reflections. The weighted *R*-factor *wR* and goodness of fit *S* are based on *F*^2^, conventional *R*-factors *R* are based on *F*, with *F* set to zero for negative *F*^2^. The threshold expression of *F*^2^ > σ(*F*^2^) is used only for calculating *R*-factors(gt) *etc*. and is not relevant to the choice of reflections for refinement. *R*-factors based on *F*^2^ are statistically about twice as large as those based on *F*, and *R*- factors based on ALL data will be even larger.

### Fractional atomic coordinates and isotropic or equivalent isotropic displacement parameters (Å^2^)

**Table:**

	*x*	*y*	*z*	*U*_iso_*/*U*_eq_	
O1	0.4334 (2)	0.6076 (3)	1.14002 (7)	0.0751 (5)	
O2	0.5923 (2)	0.6402 (2)	1.07204 (7)	0.0671 (5)	
O3	0.90619 (18)	−0.3720 (3)	0.77614 (6)	0.0682 (5)	
O4	0.86271 (17)	−0.0603 (3)	0.82460 (7)	0.0655 (5)	
Cl1	0.83872 (7)	0.86091 (8)	1.00563 (2)	0.0658 (2)	
N2	0.9571 (2)	0.2143 (3)	0.87964 (6)	0.0560 (5)	
N1	0.71450 (19)	0.4773 (2)	1.00623 (7)	0.0496 (5)	
C5	0.8366 (2)	0.3491 (3)	0.94045 (8)	0.0523 (6)	
H5	0.7886	0.2325	0.9402	0.063*	
C6	0.8069 (2)	0.4937 (3)	0.97170 (8)	0.0470 (5)	
C1	0.8754 (2)	0.6715 (3)	0.96890 (8)	0.0497 (6)	
C2	0.9714 (3)	0.6982 (3)	0.93761 (8)	0.0594 (7)	
H2A	1.0153	0.8177	0.9363	0.071*	
C3	1.0032 (3)	0.5503 (4)	0.90814 (8)	0.0602 (7)	
H3	1.0695	0.5683	0.8874	0.072*	
C4	0.9347 (2)	0.3727 (3)	0.90976 (8)	0.0519 (6)	
C7	0.6600 (2)	0.3163 (3)	1.01624 (8)	0.0509 (6)	
H7	0.6779	0.2059	0.9990	0.061*	
C8	0.5717 (2)	0.2992 (3)	1.05322 (8)	0.0474 (5)	
C9	0.5173 (3)	0.1175 (3)	1.06386 (9)	0.0572 (6)	
H9	0.5382	0.0082	1.0468	0.069*	
C10	0.4341 (3)	0.0992 (3)	1.09897 (10)	0.0633 (7)	
H10	0.3987	−0.0222	1.1057	0.076*	
C11	0.4019 (2)	0.2612 (4)	1.12488 (9)	0.0590 (6)	
H11	0.3432	0.2482	1.1483	0.071*	
C12	0.4565 (2)	0.4408 (3)	1.11604 (8)	0.0542 (6)	
C13	0.5416 (2)	0.4621 (3)	1.08000 (8)	0.0491 (6)	
C15	1.0764 (3)	0.1673 (4)	0.86971 (8)	0.0591 (6)	
H15	1.1497	0.2411	0.8819	0.071*	
C16	1.1010 (2)	0.0026 (4)	0.84010 (8)	0.0549 (6)	
C17	1.2336 (3)	−0.0564 (5)	0.83404 (10)	0.0728 (8)	
H17	1.3061	0.0163	0.8473	0.087*	
C18	1.2577 (3)	−0.2191 (5)	0.80892 (10)	0.0788 (8)	
H18	1.3463	−0.2582	0.8056	0.095*	
C19	1.1499 (3)	−0.3269 (4)	0.78828 (9)	0.0687 (7)	
H19	1.1673	−0.4366	0.7708	0.082*	
C20	1.0186 (2)	−0.2735 (4)	0.79340 (8)	0.0549 (6)	
C21	0.9926 (2)	−0.1070 (4)	0.81969 (8)	0.0521 (6)	
C14	0.3549 (3)	0.5911 (5)	1.17851 (11)	0.0893 (9)	
H14A	0.2687	0.5341	1.1687	0.134*	
H14B	0.3412	0.7181	1.1913	0.134*	
H14C	0.4013	0.5098	1.2018	0.134*	
C22	0.9258 (3)	−0.5492 (4)	0.75161 (11)	0.0839 (9)	
H22A	0.9725	−0.5221	0.7244	0.126*	
H22B	0.8394	−0.6070	0.7425	0.126*	
H22C	0.9787	−0.6377	0.7714	0.126*	
H4	0.861 (3)	0.027 (4)	0.8438 (9)	0.102 (12)*	
H2	0.643 (3)	0.620 (5)	1.0511 (9)	0.110 (13)*	

### Atomic displacement parameters (Å^2^)

**Table:**

	*U*^11^	*U*^22^	*U*^33^	*U*^12^	*U*^13^	*U*^23^
O1	0.0940 (14)	0.0583 (11)	0.0763 (12)	0.0002 (9)	0.0263 (11)	−0.0095 (9)
O2	0.0916 (14)	0.0332 (9)	0.0793 (13)	−0.0106 (9)	0.0236 (11)	−0.0061 (8)
O3	0.0633 (11)	0.0731 (12)	0.0684 (11)	−0.0013 (9)	0.0056 (9)	−0.0191 (9)
O4	0.0494 (11)	0.0733 (12)	0.0736 (12)	−0.0005 (9)	0.0040 (9)	−0.0192 (10)
Cl1	0.0819 (5)	0.0395 (3)	0.0753 (4)	−0.0074 (3)	0.0026 (3)	−0.0034 (3)
N2	0.0637 (14)	0.0580 (12)	0.0462 (11)	−0.0071 (10)	0.0044 (10)	−0.0001 (10)
N1	0.0562 (12)	0.0357 (10)	0.0563 (11)	−0.0062 (8)	0.0013 (10)	0.0008 (8)
C5	0.0593 (15)	0.0426 (12)	0.0538 (13)	−0.0104 (11)	−0.0028 (12)	0.0013 (11)
C6	0.0521 (14)	0.0405 (12)	0.0471 (13)	−0.0022 (10)	−0.0045 (11)	0.0041 (10)
C1	0.0610 (15)	0.0372 (12)	0.0490 (13)	−0.0042 (10)	−0.0064 (11)	0.0058 (10)
C2	0.0772 (17)	0.0457 (13)	0.0542 (14)	−0.0173 (12)	−0.0012 (13)	0.0103 (12)
C3	0.0732 (17)	0.0589 (15)	0.0488 (14)	−0.0128 (13)	0.0062 (12)	0.0096 (12)
C4	0.0626 (15)	0.0505 (13)	0.0419 (12)	−0.0081 (11)	−0.0005 (11)	0.0032 (11)
C7	0.0577 (14)	0.0345 (12)	0.0599 (14)	0.0004 (10)	0.0015 (12)	−0.0025 (10)
C8	0.0480 (13)	0.0359 (11)	0.0572 (14)	−0.0027 (9)	−0.0013 (11)	0.0029 (10)
C9	0.0669 (16)	0.0373 (12)	0.0669 (16)	−0.0074 (11)	0.0029 (13)	0.0001 (11)
C10	0.0705 (18)	0.0450 (14)	0.0734 (17)	−0.0167 (12)	−0.0005 (14)	0.0080 (12)
C11	0.0537 (15)	0.0626 (16)	0.0606 (15)	−0.0073 (12)	0.0044 (12)	0.0092 (13)
C12	0.0563 (15)	0.0475 (13)	0.0581 (15)	0.0012 (11)	0.0008 (12)	−0.0016 (12)
C13	0.0527 (14)	0.0352 (12)	0.0586 (14)	−0.0027 (10)	0.0001 (12)	0.0020 (10)
C15	0.0621 (17)	0.0682 (16)	0.0466 (13)	−0.0132 (13)	0.0011 (12)	0.0046 (12)
C16	0.0527 (15)	0.0685 (16)	0.0436 (13)	−0.0057 (12)	0.0045 (11)	0.0049 (12)
C17	0.0521 (17)	0.099 (2)	0.0667 (17)	−0.0091 (15)	0.0034 (14)	−0.0022 (17)
C18	0.0530 (17)	0.108 (2)	0.0767 (19)	0.0087 (16)	0.0120 (15)	−0.0070 (19)
C19	0.0680 (18)	0.0807 (19)	0.0584 (16)	0.0079 (15)	0.0111 (14)	−0.0029 (14)
C20	0.0537 (15)	0.0703 (16)	0.0409 (12)	0.0011 (13)	0.0057 (11)	0.0028 (12)
C21	0.0479 (14)	0.0653 (15)	0.0433 (12)	0.0004 (12)	0.0057 (11)	0.0039 (11)
C14	0.101 (2)	0.092 (2)	0.078 (2)	0.0127 (19)	0.0249 (18)	−0.0081 (17)
C22	0.089 (2)	0.080 (2)	0.082 (2)	0.0068 (17)	−0.0002 (17)	−0.0270 (17)

### Geometric parameters (Å, °)

**Table:**

O1—C12	1.366 (3)	C8—C13	1.403 (3)
O1—C14	1.410 (3)	C9—C10	1.361 (4)
O2—C13	1.348 (3)	C9—H9	0.9300
O2—H2	0.83 (3)	C10—C11	1.390 (4)
O3—C20	1.361 (3)	C10—H10	0.9300
O3—C22	1.427 (3)	C11—C12	1.378 (3)
O4—C21	1.343 (3)	C11—H11	0.9300
O4—H4	0.82 (2)	C12—C13	1.397 (3)
Cl1—C1	1.733 (2)	C15—C16	1.448 (3)
N2—C15	1.279 (3)	C15—H15	0.9300
N2—C4	1.419 (3)	C16—C17	1.398 (4)
N1—C7	1.273 (3)	C16—C21	1.401 (3)
N1—C6	1.410 (3)	C17—C18	1.362 (4)
C5—C4	1.376 (3)	C17—H17	0.9300
C5—C6	1.388 (3)	C18—C19	1.391 (4)
C5—H5	0.9300	C18—H18	0.9300
C6—C1	1.401 (3)	C19—C20	1.370 (4)
C1—C2	1.375 (3)	C19—H19	0.9300
C2—C3	1.377 (3)	C20—C21	1.406 (3)
C2—H2A	0.9300	C14—H14A	0.9600
C3—C4	1.397 (3)	C14—H14B	0.9600
C3—H3	0.9300	C14—H14C	0.9600
C7—C8	1.438 (3)	C22—H22A	0.9600
C7—H7	0.9300	C22—H22B	0.9600
C8—C9	1.402 (3)	C22—H22C	0.9600
			
C12—O1—C14	117.2 (2)	O1—C12—C11	124.8 (2)
C13—O2—H2	103 (2)	O1—C12—C13	115.1 (2)
C20—O3—C22	117.6 (2)	C11—C12—C13	120.0 (2)
C21—O4—H4	109 (2)	O2—C13—C12	118.4 (2)
C15—N2—C4	121.5 (2)	O2—C13—C8	122.0 (2)
C7—N1—C6	122.7 (2)	C12—C13—C8	119.6 (2)
C4—C5—C6	122.1 (2)	N2—C15—C16	122.1 (2)
C4—C5—H5	119.0	N2—C15—H15	119.0
C6—C5—H5	119.0	C16—C15—H15	119.0
C5—C6—C1	117.1 (2)	C17—C16—C21	119.2 (2)
C5—C6—N1	125.8 (2)	C17—C16—C15	120.3 (2)
C1—C6—N1	117.1 (2)	C21—C16—C15	120.4 (2)
C2—C1—C6	121.2 (2)	C18—C17—C16	120.7 (3)
C2—C1—Cl1	119.44 (17)	C18—C17—H17	119.7
C6—C1—Cl1	119.39 (19)	C16—C17—H17	119.7
C1—C2—C3	120.8 (2)	C17—C18—C19	120.1 (3)
C1—C2—H2A	119.6	C17—C18—H18	120.0
C3—C2—H2A	119.6	C19—C18—H18	120.0
C2—C3—C4	119.1 (2)	C20—C19—C18	120.9 (3)
C2—C3—H3	120.5	C20—C19—H19	119.6
C4—C3—H3	120.5	C18—C19—H19	119.6
C5—C4—C3	119.6 (2)	O3—C20—C19	125.7 (2)
C5—C4—N2	117.2 (2)	O3—C20—C21	114.8 (2)
C3—C4—N2	123.2 (2)	C19—C20—C21	119.5 (2)
N1—C7—C8	122.2 (2)	O4—C21—C16	122.3 (2)
N1—C7—H7	118.9	O4—C21—C20	118.0 (2)
C8—C7—H7	118.9	C16—C21—C20	119.7 (2)
C9—C8—C13	119.0 (2)	O1—C14—H14A	109.5
C9—C8—C7	120.2 (2)	O1—C14—H14B	109.5
C13—C8—C7	120.8 (2)	H14A—C14—H14B	109.5
C10—C9—C8	120.7 (2)	O1—C14—H14C	109.5
C10—C9—H9	119.6	H14A—C14—H14C	109.5
C8—C9—H9	119.6	H14B—C14—H14C	109.5
C9—C10—C11	120.3 (2)	O3—C22—H22A	109.5
C9—C10—H10	119.8	O3—C22—H22B	109.5
C11—C10—H10	119.8	H22A—C22—H22B	109.5
C12—C11—C10	120.3 (2)	O3—C22—H22C	109.5
C12—C11—H11	119.8	H22A—C22—H22C	109.5
C10—C11—H11	119.8	H22B—C22—H22C	109.5
			
C4—C5—C6—C1	3.9 (3)	O1—C12—C13—O2	−0.1 (3)
C4—C5—C6—N1	−175.7 (2)	C11—C12—C13—O2	179.4 (2)
C7—N1—C6—C5	9.1 (3)	O1—C12—C13—C8	−179.99 (19)
C7—N1—C6—C1	−170.4 (2)	C11—C12—C13—C8	−0.5 (3)
C5—C6—C1—C2	−2.3 (3)	C9—C8—C13—O2	179.0 (2)
N1—C6—C1—C2	177.3 (2)	C7—C8—C13—O2	0.8 (3)
C5—C6—C1—Cl1	177.84 (16)	C9—C8—C13—C12	−1.1 (3)
N1—C6—C1—Cl1	−2.5 (3)	C7—C8—C13—C12	−179.4 (2)
C6—C1—C2—C3	−0.2 (4)	C4—N2—C15—C16	178.2 (2)
Cl1—C1—C2—C3	179.66 (18)	N2—C15—C16—C17	−173.3 (2)
C1—C2—C3—C4	1.2 (4)	N2—C15—C16—C21	2.5 (4)
C6—C5—C4—C3	−3.0 (3)	C21—C16—C17—C18	−0.5 (4)
C6—C5—C4—N2	179.5 (2)	C15—C16—C17—C18	175.4 (3)
C2—C3—C4—C5	0.3 (4)	C16—C17—C18—C19	1.1 (4)
C2—C3—C4—N2	177.6 (2)	C17—C18—C19—C20	−1.0 (4)
C15—N2—C4—C5	−140.9 (2)	C22—O3—C20—C19	1.8 (4)
C15—N2—C4—C3	41.7 (3)	C22—O3—C20—C21	−176.1 (2)
C6—N1—C7—C8	176.54 (19)	C18—C19—C20—O3	−177.6 (2)
N1—C7—C8—C9	−178.1 (2)	C18—C19—C20—C21	0.3 (4)
N1—C7—C8—C13	0.1 (3)	C17—C16—C21—O4	179.1 (2)
C13—C8—C9—C10	1.4 (3)	C15—C16—C21—O4	3.3 (3)
C7—C8—C9—C10	179.6 (2)	C17—C16—C21—C20	−0.2 (3)
C8—C9—C10—C11	0.0 (4)	C15—C16—C21—C20	−176.1 (2)
C9—C10—C11—C12	−1.6 (4)	O3—C20—C21—O4	−0.9 (3)
C14—O1—C12—C11	4.3 (4)	C19—C20—C21—O4	−179.0 (2)
C14—O1—C12—C13	−176.2 (2)	O3—C20—C21—C16	178.4 (2)
C10—C11—C12—O1	−178.7 (2)	C19—C20—C21—C16	0.3 (3)
C10—C11—C12—C13	1.9 (4)		

### Hydrogen-bond geometry (Å, °)

**Table:**

*D*—H···*A*	*D*—H	H···*A*	*D*···*A*	*D*—H···*A*
O2—H2···N1	0.83 (3)	1.81 (3)	2.586 (3)	155 (3)
O4—H4···N2	0.82 (3)	1.86 (3)	2.588 (3)	148 (3)
C11—H11···O4^i^	0.93	2.59	3.392 (3)	145
C3—H3···Cg3^ii^	0.93	2.89	3.635 (3)	138

Symmetry codes:  (i) −*x*+1, −*y*, −*z*+2; (ii) *x*, *y*+1, *z*.
